# Quinoxaline derivatives as attractive electron-transporting materials

**DOI:** 10.3762/bjoc.19.124

**Published:** 2023-11-09

**Authors:** Zeeshan Abid, Liaqat Ali, Sughra Gulzar, Faiza Wahad, Raja Shahid Ashraf, Christian B Nielsen

**Affiliations:** 1 Institute of Chemical Sciences, Department of Chemistry, Government College University, Lahore, Pakistan; 2 Department of Chemistry, Queen Mary University of London, London, United Kingdom

**Keywords:** electron transport materials, non-fullerene acceptors, n-type semiconductors, organic electronics, quinoxalines

## Abstract

This review article provides a comprehensive overview of recent advancements in electron transport materials derived from quinoxaline, along with their applications in various electronic devices. We focus on their utilization in organic solar cells (OSCs), dye-sensitized solar cells (DSSCs), organic field-effect transistors (OFETs), organic-light emitting diodes (OLEDs) and other organic electronic technologies. Notably, the potential of quinoxaline derivatives as non-fullerene acceptors in OSCs, auxiliary acceptors and bridging materials in DSSCs, and n-type semiconductors in transistor devices is discussed in detail. Additionally, their significance as thermally activated delayed fluorescence emitters and chromophores for OLEDs, sensors and electrochromic devices is explored. The review emphasizes the remarkable characteristics and versatility of quinoxaline derivatives in electron transport applications. Furthermore, ongoing research efforts aimed at enhancing their performance and addressing key challenges in various applications are presented.

## Introduction

Organic semiconductors have emerged as a fascinating class of materials with significant implications for numerous scientific disciplines, including electronics, photonics, and energy conversion. These materials, composed of carbon-based molecules or polymers, offer remarkable flexibility, tunability, and processability compared to their inorganic counterparts [1–2]. Charge transport in organic semiconductors is a fundamental aspect that governs the performance and functionality of various organic semiconductor devices, such as organic solar cells (OSCs), organic field-effect transistors (OFETs), organic light-emitting diodes (OLEDs), and bio/chemo-sensing devices. The movement of charge carriers through these materials occurs via a complex interplay of electronic, structural, and energetic phenomena, presenting intriguing challenges and opportunities for scientific exploration [3–4].

**Figure 1:**
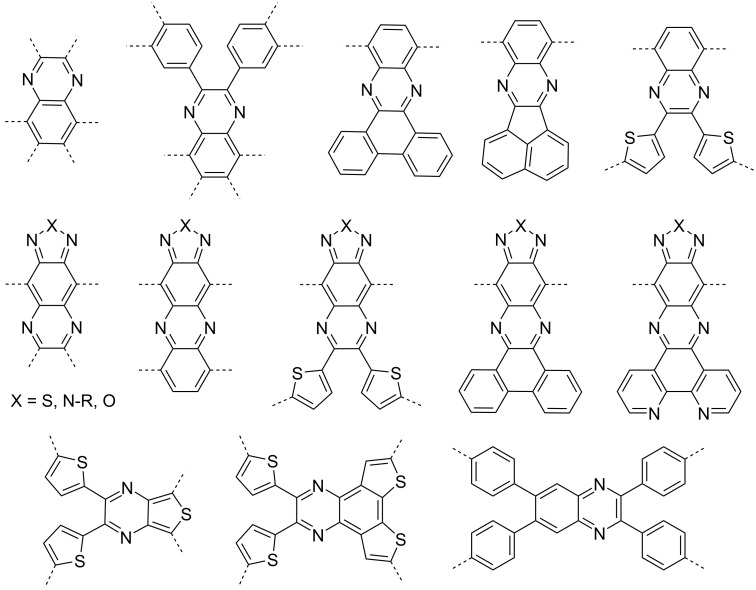
Structures of some of the most versatile Qx scaffolds; dashed lines indicate the substitution sites for core expansion.

Quinoxalines (Qxs) have emerged as a promising class of heterocyclic compounds for charge transport applications, owing to two crucial factors. Firstly, their structural diversity enables precise customization of molecular structures, allowing for fine-tuning of their properties and optimization of performance for specific applications. Secondly, the feasibility of synthesizing quinoxalines contributes immensely to their appeal. Qxs can be readily prepared through simple condensation reactions, enabling convenient experimental studies and cost-effective bulk production [5]. The availability of inexpensive and accessible starting materials further enhances the practicality and commercial viability of Qxs for charge transport applications [6–10]. Figure 1 shows a few Qx scaffolds used in development of Qx derivatives.

The utilization of Qxs as a charge transporting material, whether as a hole transport material (HTM) or an electron transport material (ETM), is largely dependent on its functionalization. Although the Qx material has primarily been recognized for its effectiveness in hole transport, several studies have unveiled its significant potential as an ETM [6–7,11–12], exhibiting desirable characteristics such as high electron mobility and efficient charge transfer. In particular, Qx derivatives find use as non-fullerene acceptors (NFAs) in OSCs and as essential building blocks in sensitizers for DSSCs. The significance of Qx extends beyond to thermally activated delayed fluorescence (TADF) emitters and chromophores in the development of organic light-emitting diodes (OLEDs), sensors, and electrochromic devices [8,13–14].

In this review, we have comprehensively examined the recent advancements of Qx-derived ETMs in various applications within the organic semiconductor device field over the past five to six years. Furthermore, we have briefly discussed the integration of Qx derivatives into relevant materials to enhance electron transport. The review also sheds light on future research directions and potential challenges in this area, emphasizing the importance of further exploration and innovation. Overall, this review presents a first detailed account of the electron transport properties of Qx derivatives in recent times, offering valuable insights into their potential as promising ETMs.

## Review

### Quinoxalines as polymer acceptors

The development of efficient and high-performing polymer materials based on Qxs is of significant interest in the field of organic electronics. A compelling indication of this potential is the remarkable achievements made by a relatively simple polymer, poly[(thiophene)-*alt*-(6,7-difluoro-2-(2-hexyldecyloxy)quinoxaline)] (PTQ10). PTQ10 has demonstrated impressive power conversion efficiencies (PCEs) of over 12% in polymer solar cells (PCS) when paired with the IDT acceptor [15], over 16% with the Y6 acceptor [16–17] and a champion PCE of 21.2% in perovskite solar cells [18]. This outstanding performance is attributed to the versatile and tunable nature of the Qx moiety, wherein researchers substituted alkoxy chains to enhance solubility and difluoro groups to lower the highest occupied molecular orbital (HOMO) energy level.

**Figure 2:**
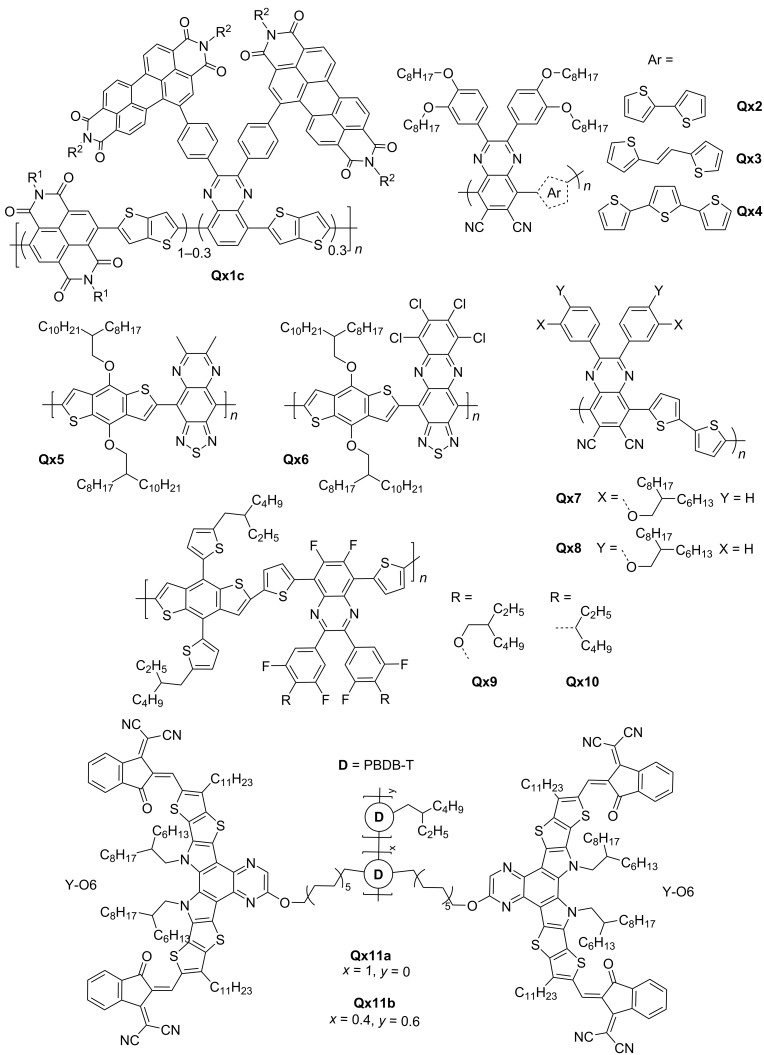
Qx-derived polymer acceptors.

Qx-derived polymer acceptors have witnessed significant progress in recent years, driven by a contextual understanding of the major issues hindering their performance as electron acceptors. Researchers have focused on improving multi-dimensional electron transport, enhancing electron injection, addressing stability concerns, optimizing side chain engineering, and refining fabrication processes. One notable study by Wang et al. demonstrated the importance of designing molecular structures that can overcome aggregation-related limitations and enhance charge transport properties in all-polymer solar cells (all-PSCs). The team incorporated twisted perylenediimide units into the polymeric backbone of (naphthalenediimide/quinoxaline)thieno[3,2-*b*]thiophene in different ratios to produce three copolymers, **Qx1a**, **Qx1b** and **Qx1c** (Figure 2). This strategy reduced aggregation and improved the performance and stability of copolymers, with **Qx1c** achieving a PCE of 4.81% with PTB7-Th donor in an all-PSC device. Moreover, the broadened absorption band indicated an expanded spectral response, suggesting potential for harvesting a wider range of photons [19].

You and co-workers demonstrated the effectiveness of introducing electron-withdrawing cyanide (CN) groups at the 6- and 7-positions of the Qx moiety, named QxCN to address various concerns related to charge transport. The copolymers, **Qx2**, **Qx3**, and **Qx4**, formed by combining QxCN with different aryl groups showed down-shifting of lowest unoccupied molecular orbital (LUMO) levels and enhanced electron injection and transport. Furthermore, the dipole moment introduced by the CN groups improved charge separation by reducing Coulomb attraction and exciton binding energy. The resulting enhancement in exciton dissociation and reduced charge recombination contribute to the improved performance of polymer acceptors, especially **Qx2** which achieved PCE of 5.32% with PBDB-T donor in an all-PSC device [20]. In a recent study by Eedugurala et al., a terachlorobenzene ring were fused to the [1,2,5]thiadiazolo[3,4-*g*]quinoxaline unit in the polymer backbone of **Qx6** which enhanced the stability of the polymer and led to its high-spin configuration compared to the analogous material **Qx5** featuring 6,7-dimethyl-[1,2,5]thiadiazolo[3,4-*g*]quinoxaline in its polymer backbone. The transition from a closed-shell aromatic state to a high-spin quinoidal form resulted in favorable changes in the bandgap, electron affinity, and delocalization of spin density. These changes have the potential to improve charge transport and efficient charge separation in all-PSCs such as switching from p-type dominated behaviour of polymer **Qx5** to n-type dominated behaviour of Q6, with no off state due to presence of free charge carriers in the latter case [21].

Besides optimizing polymer structures, side chain engineering, introduction of electron-withdrawing terminal acceptor units, and careful selection of solvents and annealing processes have also been demonstrated as potential solutions to improve charge transport and refine the device fabrication processes. You et al. found that the position of alkoxy side chains on the pendant benzene rings significantly influenced the performance of **Qx2** acceptors. Three variants of **Qx2**, i.e., **Qx7** and **Qx8** were synthesized with alkoxy side chains located at the *meta* and *para* positions of the pendant benzene rings. **Qx7** exhibited efficient exciton dissociation, good electron-transporting ability, and a PCE of 5.07% in an all-PSC device with the PBDB-T donor, whereas **Qx8** showed poor charge transport, severe charge recombination, and a PCE of 1.62%. This highlighted the significance of side chain engineering in achieving high-performance polymer acceptors [22].

In addition to the importance of side-chain modification, the study by Zhou et al. showcases the importance of solvent choice and annealing techniques in optimizing the performance of all-PSCs using **Qx9** and **Qx10**. While both polymers served as donors in all-PSC devices, the study primarily focused on side chain engineering of the electron-deficient Qx unit. The combination of thermal annealing treatment and the use of THF as a non-halogenated solvent led to improvements in photovoltaic performance and charge carrier transport. Additionally, the impact of side chain modification on device characteristics, such as lower HOMO and higher circuit voltage (*V*_oc_), underscored the influence of the molecular structure [23].

A recent development by Liang et al. introduced Qx-derived double-cable conjugated polymers as a promising approach for improving the performance of single-component-OSCs (SCOSCs). They replaced the traditional benzothiadiazole core of Y-series acceptor with the Qx moiety (Y-O6) and copolymerized it with the PBDB-T donor in two ratios to give **Qx11a** and **Qx11b**. This approach enhanced charge transport and nanophase separation, resulting in a record PCE of 13.02% in an SCOSC device made of **Qx11b** with diluted YO6 component [24].

Table 1 enlists the photovoltaic device performance of recently reported Qx materials. The structures of reviewed NFAs are drawn in Figure 2. The reviewed findings highlight that, despite limitations in the device performance, Qx derivatives possess significant potential for improvement. With further advancements and optimization, Qx polymer acceptors are expected to evolve into high-performance materials for organic electronics.

**Table 1:** Photovoltaic performance of Qx-derived polymer acceptors in PSCs.

Active layer	*V*_oc_ (mV)	*J*_sc_ (mA/cm^3^)	FF (%)	PCE (%)	Ref.

PTB7-Th:**Qx1a**	0.8	10.58	50.5	4.27	[19]
PTB7-Th:**Qx1b**	0.81	11.11	51.28	4.61	
PTB7-Th:**Qx1c**	0.82	11.72	50.27	4.81	
PBDB-T:**Qx2**	1.02	10.25	0.48	5.02	[20]
PBDB-T:**Qx3**	1	7.83	0.45	3.54	
PBDB-T:**Qx4**	1.03	2.16	0.26	0.58	
PBDB-T:**Qx2**	1	10.76	0.48	5.18	[22]
PBDB-T:**Qx7**	0.95	11.82	0.45	5.07	
PBDB-T:**Qx8**	0.98	5.16	0.32	1.62	
**Qx11a**	0.91	9.46	0.3	2.75	[24]
**Qx11b**	0.9	22.24	0.65	13.02	

### Quinoxalines as NFAs

**Figure 3:**
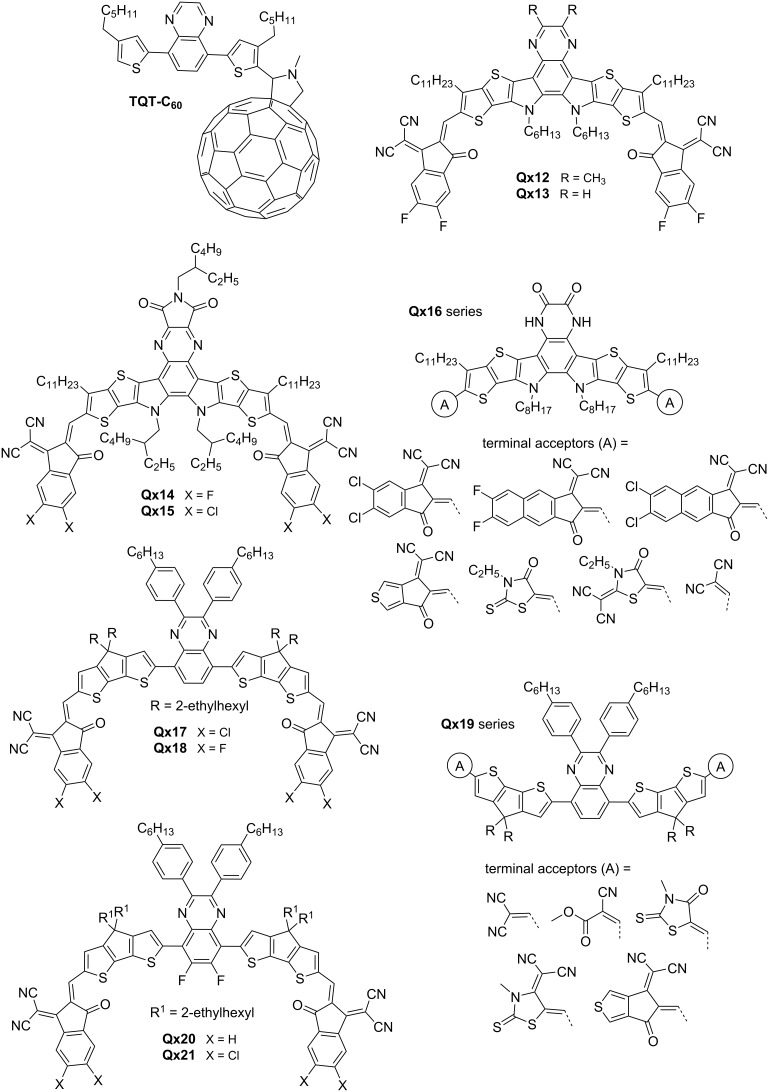
Qx-derived small molecule NFAs.

Fullerene acceptors have long dominated OSCs until the emergence of NFAs; nonetheless, researchers have attempted to improve fullerenes and address their limitations, as demonstrated by Elavarasan and colleagues. Their team synthesized **TQT-C_60_** and anchored it to fullerene molecules (Figure 3) to prevent aggregation during thermal aging. The researchers found that **TQT-C_60_** demonstrated enhanced morphological stability and thermal resistance compared to the phenyl-C61-butyric acid methyl ester (PCBM) acceptor when used in bulk heterojunction polymer solar cell devices with poly(3-hexylthiophene) (P3HT) as the donor material. This improvement was attributed to the anchoring effect of the bulkier groups present in **TQT-C_60_**, which hindered the movement and aggregation of fullerene molecules within the polymer matrix [25].

Small molecule NFAs have significantly advanced the field of OSCs. Notably, the fused-ring electron acceptors (FREAs) have exhibited exceptional promise, heralding a new era of possibilities for OSC technology. In 2019, Yuan et al. reported on a new class of FREAs called Y6. Y6 utilizes a ladder-type electron-deficient core-based central fused ring (dithienothiophen[3.2-*b*]pyrrolobenzothiadiazole) and achieved a remarkable efficiency of 16% in OSCs [26]. Building upon this breakthrough, Liu et al. blended Y6 with their polymer D18, resulting in an efficiency of 18% and marking a significant advancement in OSC research [27].

Zhou and co-workers synthesized Y6-type NFA acceptors, **Qx12** and **Qx13**, by substituting Y6’s benzothiadiazole ring with a Qx moiety. **Qx13** exhibited a stronger π–π interaction compared to **Qx12**, which facilitated enhanced electron hopping and reduced geminate recombination. **Qx12** and **Qx13** achieved remarkable PCEs of 13.31% and 16.64%, respectively, with PBDB-TF donor in OSC devices [28]. Zhu et al. reported a modification in **Qx13** by incorporating an imide-functionalized Qx moiety in its core and end-capping groups with fluorinated or chlorinated compounds, producing **Qx14** and **Qx15**, respectively. This modification aimed to improve the device performance by enhancing aggregation control and optimizing the open-circuit voltage. The introduction of functional groups provided a strategic means to tailor the molecular structure, resulting in improved photovoltaic properties and overall PCEs (12.12–13.3%) [29]. Researchers have explored Y6 derivatives for hydrogen production. Zhang and co-workers synthesized a two-dimensional polycyclic material by merging two Y6 molecules with a Qx unit. They blended the newly formed compound with the donor polymer PM6 to create BHJ nanoparticles and employed it in the hydrogen evolution reaction. This approach substantially reduced trap density, increasing the hydrogen evolution rate by 2–3 times compared to conventional inorganic/organic hybrid photocatalysts [30].

Computational chemistry offers a cost-effective and time-efficient means of screening and selecting promising candidates for experimental exploration. Bhattacharya et al. employed density functional theory (DFT) approach to explore structural modulation for tuning the optoelectronic properties of **Qx13**. Their designed molecule series, **Qx16**, featured a 1,4-dihydro-2,3-quinoxalinedione core and different terminal acceptor units. The modified NFAs demonstrated visible and near-infrared absorption as well as good electron mobility, suggesting their potential for experimental exploration in (OCSs) [31].

While FREAs are currently at the forefront and delivering great PCE, unfused electron acceptors have continued to garner considerable attention. Chang and co-workers focused on enhancing the aggregation and crystallinity of unfused Qx acceptors, **Qx17** and **Qx18**, containing a Qx core and different halogenated end groups. Both compounds exhibited good coplanarity through intramolecular interactions, narrow bandgaps, broad absorption in the NIR region and PCEs above 10% [32]. Ayub et al. decorated the central donor–acceptor–donor unit of **Qx17** and **Qx18** with five new terminal end groups, resulting in the **Qx19** series, and predicted their optoelectronic properties to highlight the potential of their experimental exploration [33].

**Figure 4:**
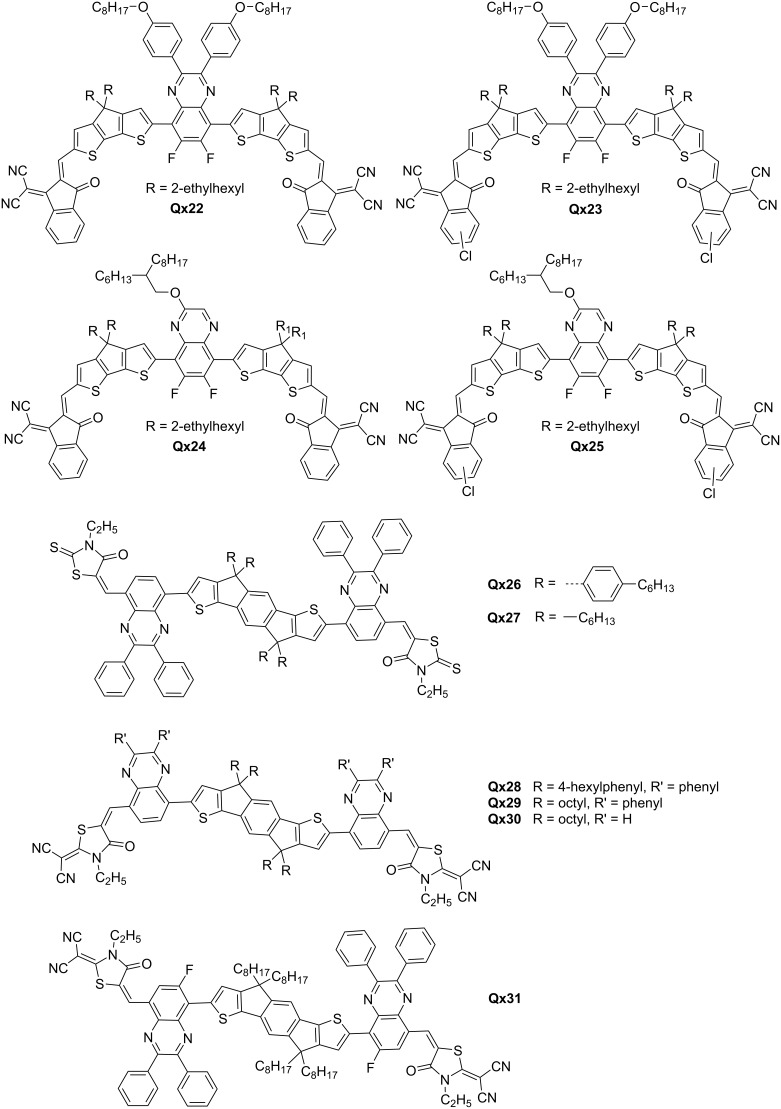
Qx-derived small molecule NFAs.

The introduction of an electron-withdrawing group has been shown to be a key strategy for enhancing intermolecular interactions and improving the optical absorption, molecular packing, and charge transport ability of NFAs. Huang et al. synthesized noncovalently fused-ring electron acceptors (NFREAs), **Qx20** and **Qx21**, featuring 6,7-difluoro-2,3-diphenylquinoxaline core. The fluorine atoms of the core formed multiple noncovalent bonds (N···H and S···F) thus improving backbone coplanarity. This approach enhanced absorption, improved carrier mobility, and reduced charge recombination of NFAs. **Qx21**, bearing chlorine atoms on the end groups as opposed to **Qx20**, showed superior optoelectronic properties and a PCE of 12.32% with PBDB-T as donor in OSC device [34]. The same group explored side-chain engineering and chlorination effects on the **Qx20** series, resulting in four new NFAs, i.e., **Qx22**–**Qx25** (Figure 4). The research revealed the strategic balance between molecular crystallinity, packing, and optical properties. **Qx24** and **Qx25**, with lower steric hindrance in the alkyl side chains, showed slightly decreased crystallinity and optical absorption but a shorter π–π stacking distance. OSC devices based on **Qx23** and **Qx25** achieved the highest PCE (10.67 and 12.19%, respectively) compared to **Qx22** and **Qx24** (6.94 and 8.01%, respectively) [35].

Xiao and fellows studied the impact of side chains on molecular packing and morphology on **Qx26** and **Qx27**, employing indacenodithiophene, Qx and rhodanine as donor, acceptor and end group, respectively. The incorporation of specific side chains facilitated improved thermal stability, solubility, and broad absorption spectra (300–750 nm), narrow bandgaps (1.68–1.74 eV) and PCEs in the range of 4.03–4.81% in PSC devices [36]. The team further explored side-chain engineering (phenyl groups) and end-group modification (2-(1,1-dicyanomethylene)thiazolidin-4-one) of new NFAs, i.e., **Qx28**–**Qx30**. While removal of all side chains supported a planar conformation of the molecule, it hampered phase separation, lowering short-circuit current (*J*_sc_), fill factor (FF) and PCE. Contrarily, the presence of all groups also compromised crystallinity and electron mobility. The highest PCE of 6.37% was realized for **Qx29** upon only taking away the phenyl side groups attached to the IDT units [37].

**Qx29** was used to prove that employing the same acceptor unit for both donor and acceptor is an effective approach. This strategy has been shown to be successful in achieving high *V*_oc_ for benzotriazole materials, and now it has been extended to quinoxaline materials [38]. Ji et al. introduced fluorine atoms to the Qx moiety of **Qx29**, producing a new NFA, **Qx31**, as well as the thiophene side chains of the p-type polymer PE61 to fine-tune the optoelectronic properties. The PCE of PE62:**Qx31**-based solar cells improved from 4.19 to 9.78% with a relatively high *V*_oc_ of 1.09 V, and the PE61:**Qx31**-based devices gave rise to the highest PCE of 10.45% [39].

These reports provide strong evidence that the ongoing exploration and refinement of Qx-based NFAs hold tremendous promise for the future development of efficient and scalable OSC technology. The reported molecules are represented in Figure 3 and Figure 4. Photovoltaic performance of some of the reported materials is tabulated in Table 2.

**Table 2:** Photovoltaic performance of Qx-derived NFAs in OSCs.

Active layer	*V*_oc_ (mV)	*J*_sc_ (mA/cm^3^)	FF (%)	PCE (%)	Ref.

P2F-EHp:**Qx14**	0.94	18.27	70.53	12.12	[29]
P2F-EHp:**Qx15**	0.94	19.62	72.11	13.3	
J52:**Qx17**	0.78	21.64	62.12	10.54	[32]
J52:**Qx18**	0.76	22.71	63.09	10.81	
PBDB-T:**Qx20**	0.862	16.19	56.64	7.9	[34]
PBDB-T:**Qx21**	0.782	22.91	69.01	12.32	
PBDB-T:**Qx22**	0.843	17.04	48.34	6.94	[35]
PBDB-T:**Qx23**	0.824	20.74	62.44	10.67	
PBDB-T:**Qx24**	0.893	16.74	53.55	8.01	
PBDB-T:**Qx25**	0.845	21.03	68.7	12.19	
P3HT:**Qx26**	0.99	4.83	0.67	3.2	[36]
P3HT:**Qx27**	0.96	6.41	0.71	4.37	
P3HT:**Qx28**	0.89	5.57	0.68	3.37	[37]
P3HT:**Qx29**	0.75	12.87	0.66	6.37	
P3HT:**Qx30**	0.75	0.14	0.3	0.03	
PE61:**Qx29**	1.02	12.03	64.52	8.24	[39]
PE62:**Qx29**	1.12	6.47	54.97	4.19	
PE61:**Qx31**	0.98	15.44	66.28	10.45	
PE62:**Qx31**	1.09	12.53	68.96	9.78	

### Quinoxalines as auxiliary acceptors and π-bridges

Qx derivatives are highly attractive auxiliary acceptor and bridging materials for DSSCs. Their strong electron-accepting ability enables efficient electron injection and charge collection, while their extended conjugation enhances light absorption across a broad spectrum. Qx’s unique structure promotes effective incorporation into the dye-sensitized layer, ensuring good intermolecular connectivity and facilitating electron transport. In addition, they enable efficient electron transfer and increased conjugation when acting as efficient π-bridge.

**Figure 5:**
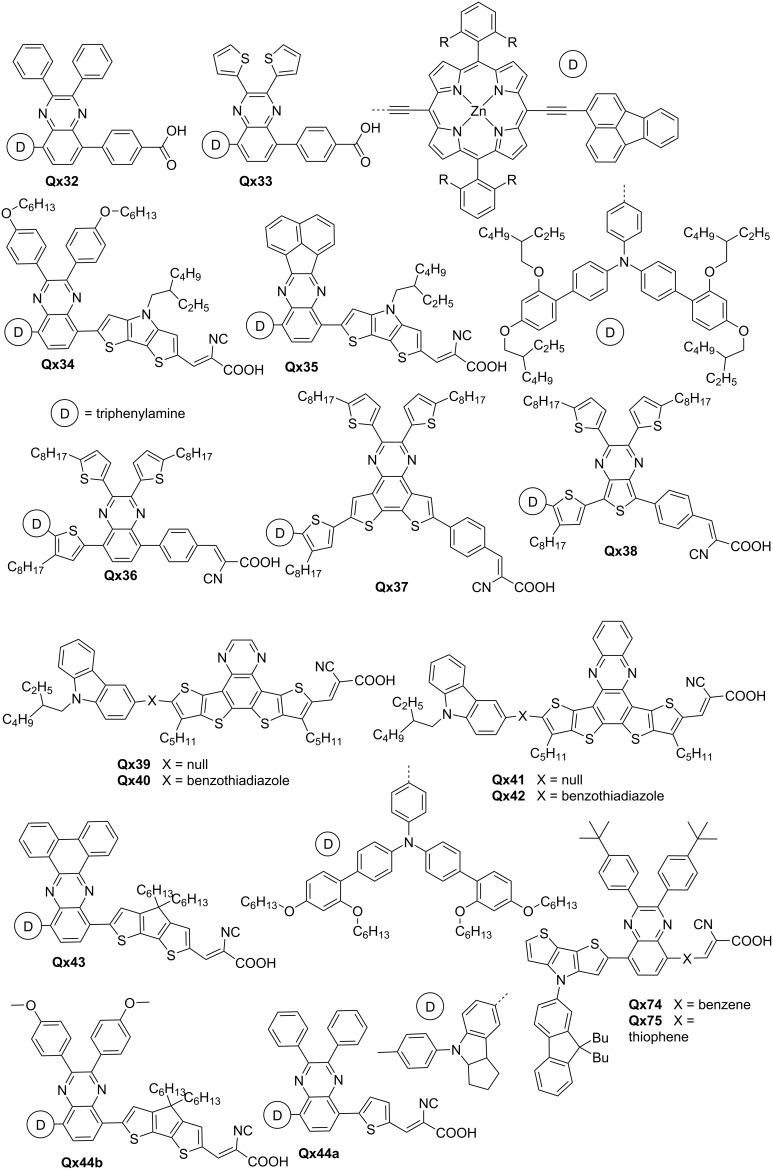
Dyes and sensitizers based on Qx auxiliary acceptors or bridging units.

Krishna et al. demonstrated the significance of Qx derivatives, 2,3-diphenylquinoxaline (DPQ), and 2,3-di(thiophen-2-yl)quinoxaline as auxiliary acceptors by effectively improving the electron injection process in **Qx32** and **Qx33** (Figure 5). The charge transfer efficiency and device performance of **Qx33** was improved by aligning its LUMO energy level with the conduction band edge of the TiO_2_ nanoparticles. This strategic approach highlights the importance of optimizing energy level alignment for efficient charge transport in DSSCs [40]. Similarly, Grobelny et al.'s work provides valuable insights into the impact of two quinoxaline derivatives, hexyloxy-substituted diphenylquinoxaline (HPQ) and naphthalene-fused-quinoxaline (NFQ), as auxiliary acceptors on DSSC performance. The comparison between **Qx34** and **Qx35** highlights the importance of the specific quinoxaline structure. **Qx34**, incorporating HPQ as the auxiliary acceptor, demonstrated enhanced electron injection and charge collection, leading to a higher PCE of 13.2% [41].

A careful choice of auxiliary acceptor is of great importance, as highlighted by Kumar and co-workers. Their study raises a concern regarding the performance of dyes with *tert*-butyl substituted DPQ acceptors, either containing benzene (**Qx74**) or thiophene (**Qx75**) as a π-conjugation linker and their benzotriazole analogue. While the incorporation of the Qx enhances the interaction between the donor and acceptor moieties, the resulting PCE falls slightly short. This observation highlights the importance of a careful choice of auxiliary acceptors to ensure optimal device performance [42]. Godfroy and colleagues also emphasized the significance of molecular structure and backbone planarity in achieving efficient charge transport in DSSCs. Their work highlights the importance of molecular structure and backbone planarity in achieving efficient charge transport in DSSCs. The sensitizers, **Qx36** and **Qx37**, employing Qx and dithieno[3,2-*f*:2',3'-*h*]quinoxaline acceptors, respectively, showed narrower absorption spectra, thus indicating well-matched energy levels, and exhibited superior performance. However, **Qx38**, featuring a thieno[3,4-*b*]pyrazine acceptor and more quinoidal backbone, suffered from reduced electron injection and increased recombination rates [43].

The challenge associated with the coplanarity of Qx-based dyes, **Qx39**–**Qx42** and the resulting device performance were highlighted by Huang et al. The use of quinoxaline-dithienothiophene and phenazine-dithienothiophene as π-bridges with the benzothiadiazole moiety as an auxiliary group did not yield the expected improvement, potentially due to the non-coplanarity of the molecular framework. DSSCs devices exhibited PCE in the range of 5.23–7.77% with **Qx41**-based device [44]. Jiang et al.'s research showcases the potential of choosing the right Qx derivatives as efficient electron-withdrawing acceptor in DSSCs. The utilization of phenanthrene-fused-quinoxaline (PFQ) in sensitizer **Qx43** resulted in exceptional PCE of 12.5%, surpassing traditional acceptor materials such as benzothiadiazole. Additionally, the improved charge recombination and stability indicate the strategic advantage of employing the right Qx derivatives for enhanced DSSC durability [45].

Several computational studies have also been performed to design dyes for optimized performance in DSSCs [46–47]. Shi and colleagues’ quantum modeling study sheds light on the optoelectronic properties of Qx-based dyes containing DPQ (**Qx44a**) or methoxy-substituted DPQ (**Qx44b**) as π units, highlighting the importance of specific substitutions. While **Qx44b** demonstrated favorable properties such as a superior dipole moment, narrow bandgap, and red-shifted absorption, the reduced charge transfer rate presented a challenge. This analysis emphasizes the need for a delicate balance between desirable electronic properties and efficient charge transfer dynamics [48]. Arunkumar and colleagues demonstrated the utilization of indolocarbazole-Qx systems named ICZS4. The comprehensive investigation of ICZS4's optoelectronic properties highlighted its potential for high-performance DSSCs. The small energy gap, red-shifted absorption, good dye regeneration, and promising NLO properties underscored the multifunctional nature of Qx derivatives as auxiliary acceptors [49].

Figure 5 depicts molecular structures of Qx derivatives used as building blocks of dyes and sensitizers. The device properties of such prominent materials are summarized in Table 3. The findings from these studies contribute to the overall understanding and advancement of quinoxaline derivatives as attractive electron-transporting materials in DSSCs.

**Table 3:** Photovoltaic performance of Qx-containing dyes and sensitizers in DSSCs.

Qx component	*V*_oc_ (mV)	*J*_sc_ (mA/cm^3^)	FF (%)	PCE (%)	Ref.

**Qx32**	0.51	3.53	72	1.5	[40]
**Qx33**	0.56	6.28	73	2.86	
**Qx34**	1.05	16.3	77.1	13.2	[41]
**Qx35**	0.95	14.7	75	10.5	
**Qx36**	796	10.91	74	6.36	[43]
**Qx37**	828	14.11	74	8.65	
**Qx38**	537	15.49	64	5.31	
**Qx39**	661.7	12.92	75.73	6.48	[44]
**Qx40**	695	12.47	73.11	6.33	
**Qx41**	691.7	15.63	71.88	7.77	
**Qx42**	661.7	13.76	74.79	6.81	
**Qx74**	605.9	4.62	0.65	1.83	[42]
**Qx75**	598.6	4.98	0.66	1.97	

### Quinoxalines as n-type transistor materials

Qxs have also emerged as promising candidates for n-type transistor materials, offering a range of properties specifically tailored for OFET applications. The tunable properties of Qxs as n-type semiconductor materials, including high electron mobility, optimal energy levels, broad absorption spectra, and processing compatibility, position them as promising candidates for OFETs and similar electronic technologies. The incorporation of Qx derivatives into electronic devices holds great potential for achieving high-performance, energy-efficient, and scalable electronic systems in diverse fields such as energy conversion, information processing, and beyond.

**Figure 6:**
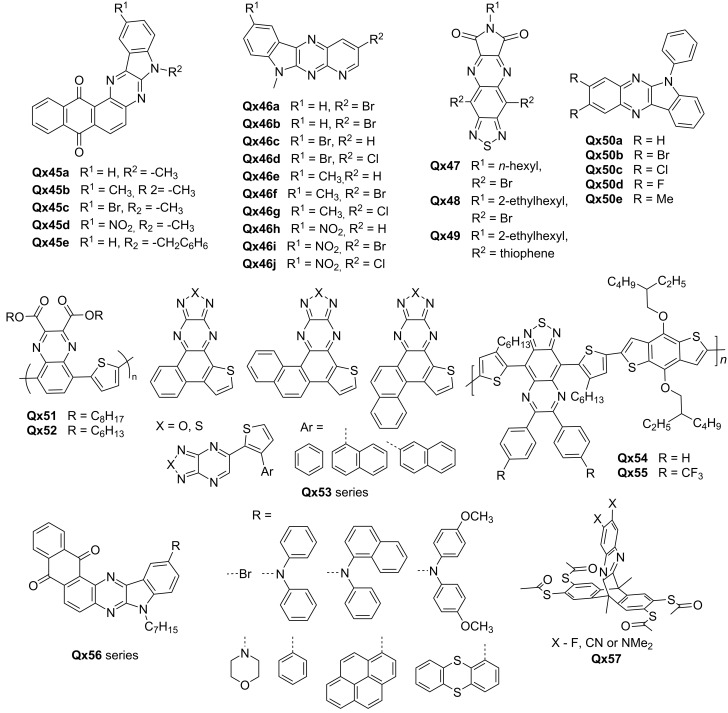
Qx-derived n-type transistor materials.

In the context of structural modification, the introduction of various functional groups into Qx derivatives has allowed for the precise control of energy levels, bandgaps, and carrier transport properties. For instance, Sharma et al. fine-tuned the intermolecular charge transfer (ICT) transitions and emission properties of **Qx45** series by incorporating electron-donating (methyl) and electron-withdrawing groups (bromo and nitro) (Figure 6). The observation of low-lying LUMO levels (−3.29 to −3.43 eV) and thermal stability in these dyes suggested their potential as efficient ETMs [50]. Similarly, Singh et al. explored the modulation of optoelectrochemical properties and thermal characteristics of pyridopyrazino[2,3-*b*]indole-based **Qx46** series with varying substituents, i.e., bromine, chlorine, methyl and nitro group. Their study revealed inbuilt ICT and aggregation-induced emission (AIE) effects, forming emissive nanoaggregates in a THF/H_2_O mixture. Altering the substituents proved as an effective approach to tune the electrochemical properties of the compounds, resulting in comparable LUMO energy levels. The products therefore hold potential as solid-state emitters and n-type materials for organic electronics [51].

Hasegawa and colleagues focused on achieving air-stable n-channel conduction by synthesizing thiadiazole-fused quinoxalineimide derivatives, **Qx47**–**Qx49**. The team optimized the molecular packing and solubility by incorporating solubilizing N-substituted alkyl chains. The resulting molecules exhibited low LUMO levels and two-dimensional carrier transport, enabling OFET performance. The moderate air-stable n-channel mobility of 0.044 cm^2^ V^−1^ s^−1^ demonstrated the suitability of these derivatives for electron transport [52]. Hayashi et al. introduced a new avenue for developing n-type *N*-phenylindoloquinoxaline (PhIQ) derivatives, shown in Figure 6 as **Qx50** series, by chemically modifying the PhIQ groups. In particular, substituents at the 2- and 3-positions of PhIQs were introduced, allowing regulation of the reduction–oxidation potentials of the compounds. The PhIQs exhibited fluorescent solvatochromism and demonstrated n-type properties due to the electronegative Qx unit [53].

Mikie et al. explored ester-functionalized quinoxalines (QEs) as building units for both p-type and n-type polymers. They synthesized two new π-conjugated polymers, **Qx51** and **Qx-52**, with low-lying HOMO (–5.5 eV) and LUMO (–3.4 eV) levels, resulting in narrow optical bandgaps (1.6 eV). In OFETs, **Qx51** exhibited hole (μ_h_) and electron (μ_e_) mobilities of 1.1 × 10^−3^ and 6.6 × 10^−4^ cm^2^ V^−1^ s^−1^, which were higher than those of **Qx52** (μ_h_ = 4.4 × 10^−4^ cm^2^ V^−1^ s^−1^, μ_e_ = 3.0 × 10^−4^ cm^2^ V^−1^ s^−1^), due to the higher crystalline nature of the earlier [54]. Recently, Ding et al. prepared a novel air-stable n-type benzothiophene endcapped azaarene (BTPQ) and its sulfonated derivative (BSPQ). By introducing nitrogen atoms and sulfonyl groups, the researchers modulated the molecular energy levels and achieved the energy level requirements of n-type semiconductors. The strategy also involved attaching triisopropylsilyl groups to the anthracene core to balance solubility and charge carrier properties. The BSPQ derivative exhibited deeper frontier orbital energy levels and enhanced electron mobility compared to the BTPQ [55].

Several reports have highlighted the development of novel synthetic routes for producing Qx derivatives with desired properties. Kvashnin et al. reported a simple yet promising strategy for designing and synthesizing n-type semiconductors. The team developed a synthetic route to produce polycyclic (hetero)aromatic compounds (**Qx53**) with a chalcogenodiazolo[3,4-*b*]pyrazine scaffold. These compounds exhibited narrow bandgaps (from 1.25 to 1.44 eV) and demonstrated n-type organic semiconductor properties [56]. Jin and co-workers focused on improving the charge-transfer characteristics of a semiconducting copolymer, benzodithiophene-thiadiazolo-quinoxaline (**Qx54**), by introducing trifluoromethyl groups to the Qx moiety (**Qx55**). This strategic modification changed the HOMO and LUMO levels, resulting in a conversion from ambipolar charge transport to n-type charge transport. The polymeric thin-film transistors (PTFTs) with **Qx54** copolymer showed ambipolar characteristics, while the PTFTs with **Qx55** copolymer exhibited only n-type charge transport [57].

Kamble et al. designed and synthesized a series of eight new indolo[2,3-*b*]naphtho[2,3-*f*]quinoxaline derivatives (**Qx56**) by incorporating an electron-accepting quinone unit on quinoxaline to achieve donor–acceptor interactions and desirable electronic properties. The compounds exhibited absorption, emission, electrochemical, and thermal properties suitable for n-type materials. Theoretical properties were also investigated using time-dependent DFT. The HOMO and LUMO energy levels of the compounds ranged from −6.51 to −6.84 eV and −3.00 to −3.30 eV, respectively. The low-lying LUMO energy levels were similar to well-known n-type materials, indicating the potential of the synthesized compounds as n-type materials in organic electronics [58].

Rohnacher et al. synthesized a tetrapodal scaffold using diazatriptycene with thiol anchors (**Qx57**) to demonstrate electrostatic dipole engineering in n-type OFETs. The scaffold was designed to enforce upright functional groups, particularly quinoxaline subunits, and utilized OFETs as prototypes to showcase the potential of self-assembled monolayer in devices. The molecular dipole and work function of gold was adjusted by using fluorine and CN as well as dimethylamino substituents on the quinoxaline. Notably, the researchers tuned the work function of gold over a range of 1.0 eV [59]. The study by You et al. quoted in the section "Quinoxalines as polymer acceptors" also fabricated OFETs using QxCN-based polymer acceptors and demonstrated unipolar n-type characteristics with moderate OFET mobilities. The well-ordered structures with tight π–π stacking in **Qx2** and **Qx3** contributed to electron mobilities greater than 1.0 × 10^−4^ cm^2^ V^−1^ s^−1^ [20]. Figure 6 shows the molecular structures of the prominent compounds exhibiting potential as n-type materials. Table 4 lists OFET device properties of devices employing Qx derivatives.

**Table 4:** OFET properties of Qx-derived OFET devices.

Materials	µ_e_ average (max) (cm^2^ V^−1^ s^−1)^	Threshold voltage (V)	*I*_on_/*I*_off_	Ref

**Qx47**	8.0 × 10^-2^ (9.0 × 10^−2^)	10.4	≈10^4^	[52]
**Qx48**	2.1 × 10^-4^ (2.7 × 10^−4^)	36.8	≈10^2^	
**Qx49**	8.0 × 10^-4^ (8.5 × 10^−4^)	43.9	≈10^3^	
**Qx51**	6.6 × 10^−4^	48	–	[54]
**Qx52**	3.0 × 10^−4^	50	–	
**Qx2**	(7.6 ± 0.4) × 10^−3^	19.9 ± 0.7	>10^3^	[20]
**Qx3**	(2.4 ± 0.1) × 10^−2^	23.1 ± 0.5	>10^3^	
**Qx4**	(7.4 ± 0.5) × 10^−3^	26.7 ± 0.4	>10^3^	

### Quinoxalines as ETL and TADF emitters

Qx derivatives have garnered substantial attention from the scientific community due to their remarkable characteristics as electron-transporting and hole-blocking layers in organic electronics [60]. Moreover, these derivatives have exhibited promising traits as thermally activated delayed fluorescence (TADF) emitters. This multifaceted nature has spurred ongoing research efforts aimed at overcoming various hurdles and enhancing the performance of Qx derivatives.

**Figure 7:**
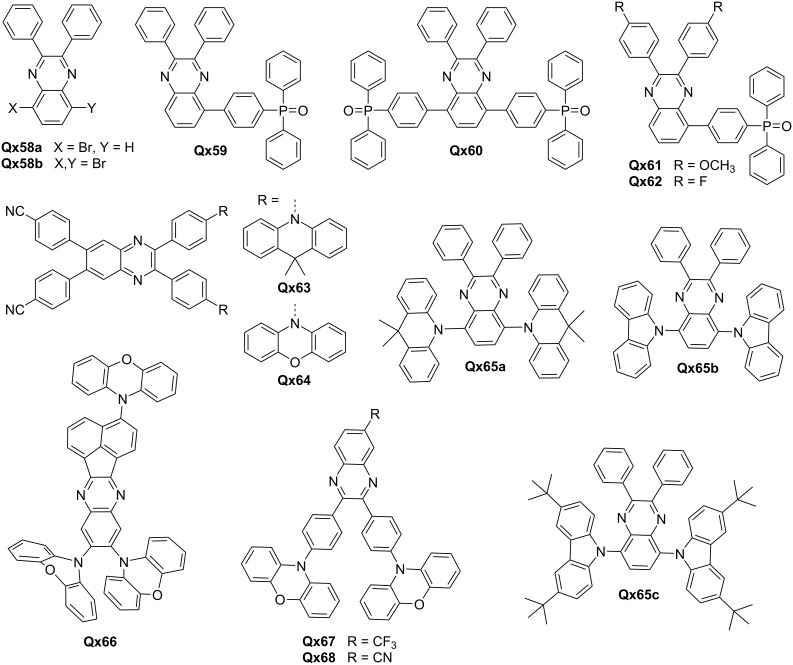
Qx-derived ETM and TADF emitters.

One key concern is the solubility of Qx derivatives in solution processing, as it affects their suitability for practical applications. Additionally, achieving efficient electron injection and extraction, as well as controlling the interfacial dipole, are crucial for improved device performance. The work by Kim et al. demonstrated the potential of Qx compounds (**Qx59** and **Qx60**) derived from **Qx58a** and **Q58b**, respectively, as solution-processable ETLs for OSCs and OLEDs (Figure 7). The incorporation of strong dipole moments in **Qx59** improves electron injection/extraction and energy level alignment, leading to enhanced device performance. The high PCE of 16.83% in OSC and excellent external quantum efficiency (EQE) of 5.00% in OLED devices highlight the efficacy of **Qx59** in facilitating efficient charge transport and emission processes [61].

To address the challenge of interfacial dipole and solubility, Lee and colleagues modified **Qx59** by introducing methoxy (**Qx61**) and fluorine (**Qx62**) groups at the 2,3-positions of the Qx ring. This modification not only facilitated easy deposition but also enhanced electron injection and transport behavior by effectively matching the band levels of the devices. The improved EQE of the OLEDs (6.12%) suggests the effectiveness of Qx derivatives as ETLs in achieving high device performance [62]. Ji et al. successfully developed TADF emitters, **Qx63** and **Qx64**, based on quinoxaline-4,4'-dicyanobenzene by manipulating the donor–acceptor conformation. The efficient TADF emission at room temperature highlighted the potential of these emitters for achieving high-performance OLEDs. The tunability of the emission peak through appropriate donor selection further demonstrates the versatility of quinoxaline derivatives in tailoring the emission properties of TADF materials. The vacuum deposited OLEDs based on **Qx63** and **Qx64** emitted yellow and red light, achieving EQEs of 17.3% and 15.6%, respectively [63].

You and co-workers reported the strategic design of a series of butterfly-shaped high-performance red/orange TADF emitters (**Qx65**). The team successfully transitioned the emission type from local excited-state to charge-transfer state by carefully tuning the molecular structure and energy levels, leading to efficient TADF. The **Qx65a**-based orange TADF OLEDs exhibit a maximum EQE of 7.4%, corresponding to a prominent contribution of 97% from the delayed fluorescence to the overall EQE [64]. Another study by Yu et al. reports the successful synthesis of a red TADF molecule, **Qx66**, based on an acenaphtho[1,2-*b*]quinoxaline acceptor. The well-separated energy levels of the molecules indicated efficient charge transfer and exciton formation within the molecule, leading to red emission. The achieved EQE of 7.4% highlighted the potential application of this Qx-based TADF emitter as dopant in red-emitting OLED devices [65].

Huang et al. developed two yellow TADF emitters, **Qx67** and **Qx68**, based on 6-(trifluoromethyl)quinoxaline or 6-cyanoquinoxaline acceptors, respectively. The small energy splitting values (0.03–0.04 eV) and long fluorescence lifetimes (5.0 μs) indicated efficient TADF processes. The utilization of these emitters in full-TADF white OLEDs, along with a sky-blue emitter, demonstrates their potential for practical applications in the lighting and display fields, with high efficiency (20.16%) and stable color rendering [66]. Gupta and co-workers designed and synthesized Y-shaped Qx derivatives with quadrupolar and tripodal arrangement. The molecules showed unique properties, including solvatochromism and AIE enhancement. These characteristics enable tunable emission and enhanced luminescence efficiency. The potential application of tripodal derivatives in white OLEDs indicates the versatility of quinoxaline-based materials in achieving diverse emission colors [67]. Figure 7 shows the prominent examples of recently reported Qx based dyes and TDAF emitters. Table 5 lists device properties and relevant information of devices employing Qx derivatives.

**Table 5:** Optoelectronic properties of Qx-derived ETL and TADF emitters.

Materials	*L*_max_(Cd/m^2^)@bias	*LE*_max_(Cd/A)@bias	EQE_max_@bias	*V*_on_ (V)	Ref.

ITO/PEDOT:PSS/SY/**Qx59**/AI	26400@10.0	14.45@5.8	5.00@5.8	2.2	[61]
ITO/PEDOT:PSS/SY/**Qx60**/AI	8660@11.0	7.73@8.0	2.71@8.0	2.2	
ITO/PEDOT:PSS/SY/**Qx59**/AI	6211@9.1	16.69@4.3	5.65@4.3	2.5	[62]
ITO/PEDOT:PSS/SY/**Qx61**/AI	10030@8.8	17.98@4.3	6.12@4.3	2.5	
ITO/PEDOT:PSS/SY/**Qx62**/AI	3723@10.5	8.76@4.8	2.94@4.8	2.5	
**Qx65**	11456	19.7	7.4	3.8	[64]
DMAC-DPS:**Qx67**	6803	48.34	18.06	2.8	[66]
DMAC-DPS:**Qx68**	6858	48.22	20.16	2.8	

### Quinoxalines as chromophores

Qx derivatives have emerged as promising chromophores due to their distinctive optical and electronic characteristics. To enhance their performance in sensors and electrochromic devices, researchers have concentrated on manipulating their electron transport properties. By tailoring the strength of electron acceptors, fine-tuning the electronic nature of functional groups, incorporating additional functionalities, and optimizing the π-conjugated backbone structure of Qxs, significant advancements have been made. These approaches effectively address key concerns and obstacles, resulting in red-shifted absorption and emission maxima, improved nonlinear optical properties, solvatochromism, mechanical responsiveness, acidofluorochromism, AIEE, and enhanced electrochromic performance.

**Figure 8:**
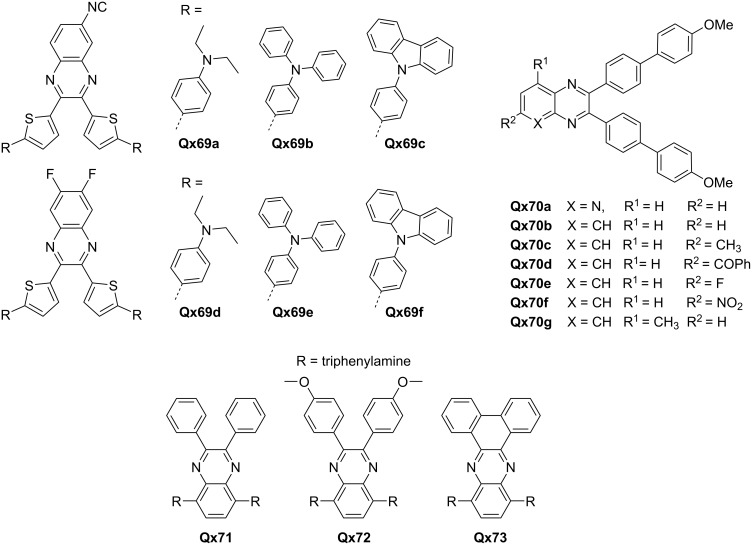
Qx-derived chromophores.

One effective approach, as demonstrated by Moshkina et al., involved introducing CN and fluorine substitutions on the Qx core to give **Qx69** (Figure 8). This modification resulted in red-shifted absorption and emission maxima, indicating the potential for tunable optical properties based on the surrounding environment. The replacement of difluoroquinoxaline with cyanoquinoxaline improved the nonlinear optical properties, highlighting the importance of tailoring electron acceptor strength. Furthermore, the ability to induce changes in emission properties through mechanical stimulation offers exciting possibilities for optomechanical applications [68].

Another notable strategy, performed by Gupta and co-workers, involves fine-tuning of the electron accepting tendency of quinoxaline by incorporating various groups with different electronic natures (**Qx70a**–**Qx70g**). This approach allowed for the modulation of chromophoric properties, including solvatochromism, acidofluorochromism, and twisted-ICT. The incorporation of a pyridopyrazine derivative further expanded the range of functionalities and enabled logic gate operations, showcasing the potential of quinoxaline-based chromophores in sensor applications and molecular logic devices [69].

In terms of electrochromic performance, the work by Fu et al. emphasized the significance of the π-conjugated backbone structure in achieving desirable electrochromic properties (**Qx71**–**Qx73**). The incorporation of twisted substituents on the backbone, as observed in polymers **Qx71** and **Qx72**, led to excellent electrochromic performance attributes. In contrast, the use of a fused electron acceptor unit in polymer **Qx73** resulted in suboptimal electrochromic performance. This highlights the strategic significance of molecular design, particularly the backbone structure, for achieving high-performance electrochromic materials with up to 70% optical contrast, <3 seconds response time, over 200 cm^2^ C^−1^ coloration efficiency and good cycle stability [70].

The molecular structures of the mentioned compounds are shown in Figure 8. These studies provide compelling evidence for the versatile applications of Qxs as ETMs in various fields, including sensors, electrochromic devices, optomechanics, and molecular logic devices.

## Conclusion

In conclusion, the reviewed studies highlight the tremendous potential of Qx-derived ETMs across various fields, including all-PSCs, OSCs, DSSCs, OFETs, and OLEDs. Their unique properties, coupled with ongoing research, open up new avenues for their utilization in various fields.

Qx derivatives have demonstrated promise in the development of gas sensors for detection, environmental monitoring, and chemical sensing. Additionally, they can be utilized for biosensing applications by leveraging their ability to absorb and emit light in the near-infrared II range. This range allows for deeper tissue penetration, reduced scattering, and minimized autofluorescence, thereby enabling enhanced sensitivity, selectivity, and accuracy in biomarker detection, physiological parameter monitoring, and disease diagnosis. Furthermore, their efficient charge transport properties make them valuable in improving energy storage and conversion systems, including batteries and supercapacitors.

The ongoing research is focused on enhancing device efficiency through the exploration of novel device architectures, interface engineering, and material modifications. It is crucial to also address the long-term stability and durability of devices that incorporate Qx-based materials. Future advancements should focus on understanding degradation mechanisms, developing effective device encapsulation strategies, and ensuring environmental compatibility to guarantee sustained performance and viability in commercial applications. Additionally, scalability and cost-effective manufacturing processes are key for the widespread adoption of Qx-based materials. To achieve this, it is important to optimize material synthesis methods, explore solution-processable routes, and develop efficient deposition techniques. These efforts will pave the way for large-scale production of electronic devices utilizing these materials.

In addition, Qx-based materials offer notable sustainability advantages. These materials possess low environmental impact and can be synthesized from abundant precursors, making them both cost-effective and environmentally friendly compared to certain inorganic semiconductor materials. Moreover, their potential in renewable energy applications aligns with the objectives of clean energy generation and reducing dependence on fossil fuels. As research progresses, we anticipate significant advancements and breakthroughs that will pave the way for a future where quinoxaline-based materials play a vital role in shaping the next generation of electronic devices and renewable energy systems.
